# Determining a Trusting Environment for Maternity Care: A Framework Based on Perspectives of Women, Communities, Service Providers, and Managers in Peri-Urban Kenya

**DOI:** 10.3389/fgwh.2022.818062

**Published:** 2022-04-21

**Authors:** Pooja Sripad, Maria W. Merritt, Deanna Kerrigan, Timothy Abuya, Charity Ndwiga, Charlotte E. Warren

**Affiliations:** ^1^Population Council, Washington, DC, United States; ^2^Department of International Health, Johns Hopkins Bloomberg School of Public Health, Baltimore, MD, United States; ^3^Johns Hopkins Berman Institute of Bioethics, Baltimore, MD, United States; ^4^Department of Prevention and Community Health, Milken Institute School of Public Health, George Washington University, Washington, DC, United States; ^5^Population Council, Nairobi, Kenya

**Keywords:** trust determinants, framework, institutional ethnography, maternity, health systems, Kenya

## Abstract

Trust in health service providers and facilities is integral to health systems accountability. Understanding determinants of trust, a relational construct, in maternity settings necessitates exploring hierarchical perspectives of users, providers, and influencers in the care environment. We used a theoretically driven qualitative approach to explore trust determinants in a maternity setting across patient-provider, inter-provider, and community-policymaker interactions and relationships in peri-urban Kenya. Focus groups (*n* = 8, *N* = 70) with women who recently gave birth (WRB), pregnant women, and male partners, and in-depth-interviews (*n* = 33) with WRB, health care providers and managers, and community health workers (CHWs) were conducted in 2013, soon after the national government's March 2013 introduction of a policy mandate for “Free Maternity Care.” We used thematic coding, memo writing, and cross-perspective triangulation to develop a multi-faceted trust determinants framework. We found that determinants of trust in a maternity setting can be broadly classified into six types of factors, where each type of factor represents a cluster of determinants that may each positively or negatively influence trust: patient, provider, health facility, community, accountability, and structural. Patient factors are prior experiences, perceived risks and harms, childbirth outcomes, and maternal health literacy. Provider factors are empathy and respect, responsiveness, and perceived capability of providers. Health facility factors are “good services” as perceived by patients, physical environment, process navigability, provider collaboration and oversight, discrimination, and corruption. Community factors are facility reputation and history, information channels, and maternal health literacy. Accountability factors are alignment of actions with expectations, adaptations to policy changes, and voice and feedback. Structural factors are institutional hierarchies and policies in the form of professional codes. Trust determinants are complex, nuanced and reflect power dynamics across relationships. Findings offer insight into socio-political maternity norms and demand a more equitable care interface between users and providers.

## Introduction

The purpose of this study is to explore what determines trust in a maternity setting. The notion of trust is implicit in discussions of maternity experiences at the health service interface, often as a critical intermediary construct between experience of care and care-seeking intentions. Increasingly, trust is globally recognized as a health systems functionality and service quality outcome (1). Because trust is relational and multidimensional, it is relevant in contexts where a range of interpersonal and systemic relations operate distinctly (2).

Trust reflects a person's belief that their expectations will be met favorably by another individual or a system in a particular time and context (3). A sense of trust may be rooted in cultural and normative perspectives, as trust reflects cooperative attitudes, mutual understanding, and the “social fabric” of a community (4). Giddens posits impersonality as key to understanding trust in health systems settings, where information flow and behaviors co-mingle in complex ways: for instance, through structural factors such as professional codes, health policies, and the distribution of clinics and hospitals (5). Impersonal trust in the maternity setting (“maternities”) goes beyond the health facility to government institutions committed to the public interest. Trust in maternities takes impersonal and interpersonal forms, is multidimensional, and is relatively unexplored (6, 7).

### Trust, Power, and Gender

Trust is integral to fiduciary care in user-provider relationships where the user's explicit vulnerability creates a power imbalance; when trust functions optimally, the provider demonstrates competence, honesty, fidelity, and non-exploitation (8). Accordingly, trust offers a lens through which to examine power and gender hierarchies within health systems (9). In the context of maternity care, female care-users may simultaneously experience sociocultural disadvantage, the burden of childbearing norms, and the social process of delivering within a power-laden health system. In many societies, childbirth is a “special event” requiring sensitivity and attention in light of women's socio-culturally prescribed roles (10). Societal and professional gender norms also affect female providers and managers, with implications for users' trust in health services.

Trust in maternity care is likely influenced by several factors closely related to those associated with women's care seeking. Studies in sub-Saharan Africa suggest that as women learn through previous experience of observed and perceived quality of health services (of any type) at facilities across different levels of a health system, trust plays a role in their active selection of where to give birth (11, 12). A woman's trust during labor and delivery is likely to be affected by her perceptions of quality of care, cumulative respectful interactions with a range of health care providers (physicians, nurse/midwives, community health workers), referral processes, and facility capacity to respond to complications safely and in a timely manner (13, 14). Psychosocial learning pathways involving trust are increasingly relevant in urban and peri-urban areas where women engage in more frequent health service interactions, are exposed to a wider range of facility options, and witness inequities in access to high-quality care (15). Beyond the dimensions of cost, distance, family dynamics, and quality that influence care seeking (16), it is important to explore trust determinants through the power relations between users, providers, managers, and stakeholders that arise within the care environment.

### Kenya: Socio-Political and Maternity Care Context

Socio-political context influences perceptions of maternity experience. For this study, conducted in 2013, Kenya's new constitution, transitioning governance structures, and free maternity policy are paramount. The 2010 Kenyan Constitution's embrace of health sector rhetoric (“accountability,” “people-centeredness,” “responsiveness,” “equity,” and “quality and access”) has implications for trust in public facilities (17). A policy of devolution transfers administrative, financial, and procedural power from national governing bodies to county level. In maternity care, devolution translates into the political restructuring of health facility management and community health leadership (18).

In addition to the shifting political environment of devolution, the national administration elected in March 2013 introduced a policy mandate for “Free Maternity Care,” placing into immediate effect universal, free-of-charge labor and delivery services in all public health facilities. The policy did not cover private or faith-based facilities (18, 19). The mandate, publicized through the national media, led almost overnight to maternity ward saturation. Increased burdens on already-overwhelmed health systems are observed in and congruent with other sub-Saharan African countries' experiences of removing maternity care-user fees (18, 20).

Disparities in maternal health status and service use in Kenya suggest variable burden and access across geographic areas and populations. A study assessing distributions of maternal death in urban poor populations found maternal mortality ratios as high as 706 deaths/100,000 live births and suggested that the majority of maternal deaths occur in facilities (21). In urban settings, despite fewer physical access barriers and greater investments in reducing out-of-pocket costs for facility delivery (22), delayed care seeking and low quality of care persist among urban and peri-urban poor populations, leading to inequitable service use and outcomes (23). In Kenya, inequities not only emerge from negative experiences motivating women to seek alternative sources of care, but also are compounded by poverty and adverse social and physical environments (24). Facility-based maternity care is typically provided by skilled providers across both public and private sectors, primarily nurse-midwives (normal deliveries) and doctors (sections), and for the poor, primarily through the government. Public facilities follow a tiered system where community health workers (CHWs) provide the first level of care via community health units, dispensaries and clinics provide the second, health centers and sub-county hospitals the third, and county and referral hospitals the fourth to sixth (25).

### Conceptual Framework

**Figure 1:**
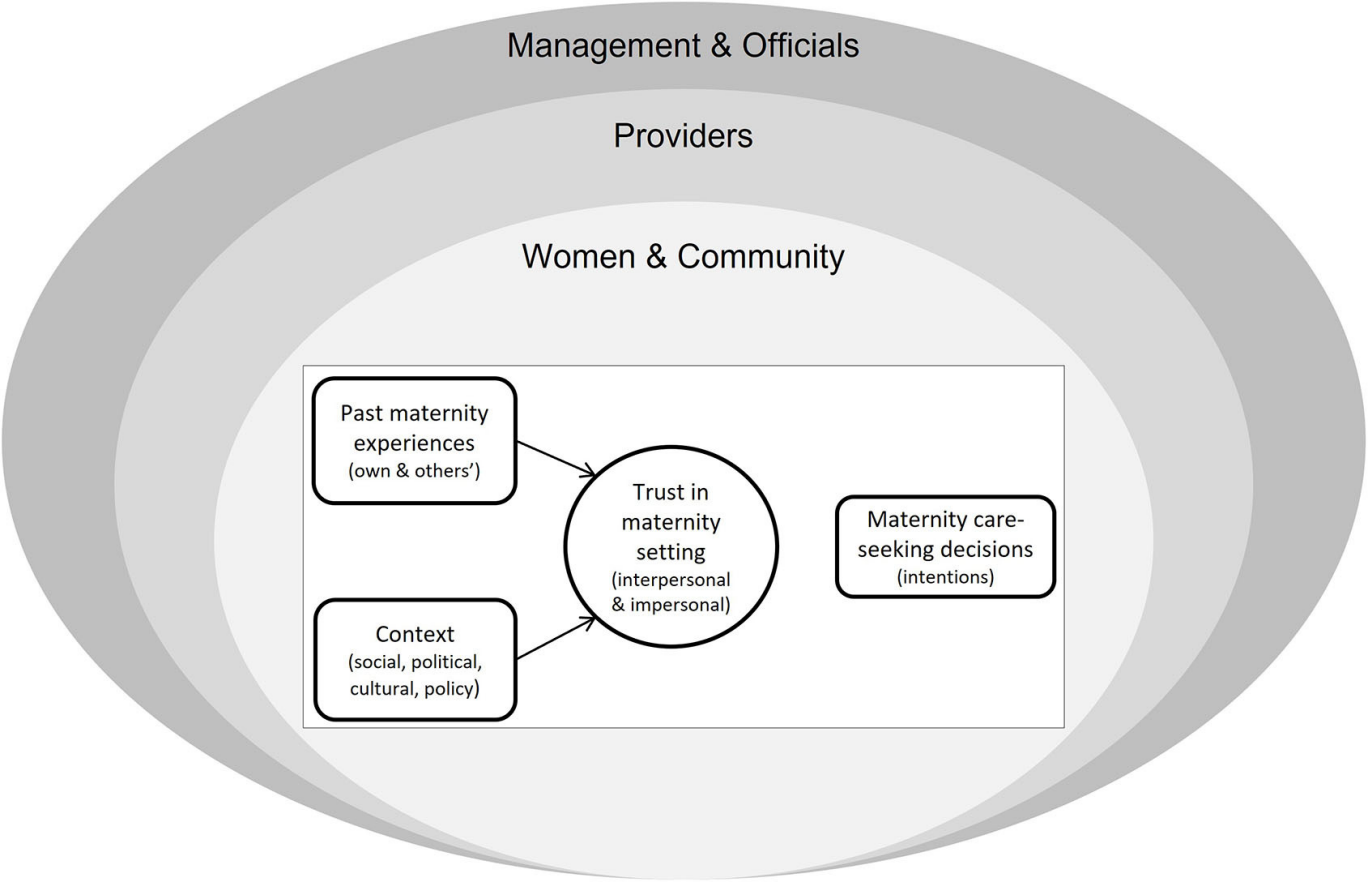
Conceptual framework.

This study's conceptual framework (Figure 1) draws on prior frameworks of trust in maternities (26) and of layered trust relationships (14). It encompasses interpersonal and impersonal trust relationships. The framework recognizes that trust not only has intrinsic value but may also be instrumental in promoting future maternity-care-seeking intentions. The concentric ovals represent the hierarchical perspectives examined in this study, from maternity care-users (women and communities) and providers to management-level actors.

## Materials and Methods

This study adopts a theoretically driven qualitative approach to explore trust determinants through perceptions of interactions and relationships among care-users, providers, and managers and policy officials. Qualitative methodology is well-suited to describing varied, nuanced trust-determining processes from individual and collective perspectives (27). Institutional ethnography with a critical gender-sensitive lens investigates power imbalances surrounding maternity experiences (28). By situating maternity experience in the socio-political discourse of devolution and free maternity in Kenya, institutional ethnography allows for understanding how institutional norms influence trust in the context of policy change. This study applies institutional ethnography by analyzing upward and downward within the hierarchical health systems structure to explore trust determinants in reference to texts (maternity policy and guidelines) and multiple standpoints. We used focus group discussions (FGDs) to study community group-normative perspectives of women and men who had recently had a child and in-depth interviews (IDIs) to study individual perspectives of women who had recently given birth, CHWs, nurse/midwives, doctors, and managers. At study design, data collection, and analysis stages, we used appreciative inquiry techniques, building on care-user, provider, and manager values to envision positive determinants of trust (29).

### Study Site

This study, nested within a larger investigation of disrespect and abuse in maternity care, the Heshima Project (2010-2014) (30), was conducted in peri-urban Central Kenya in and among communities residing around a sub-county public hospital. In 2013, when this study was conducted, 42% of women in Kenya gave birth in facilities (19). The sub-county hospital averaged 673 births per month at the time of the study and, as a referral hospital, served a catchment population of approximately 1,000,000 (31). The sub-county hospital has an administrative wing comprised of facility management and county health offices, outpatient and inpatient wards, and an operating theater; the maternity ward consists of a labor ward, delivery room for normal births, and a postnatal ward. This facility provides comprehensive emergency obstetric and newborn care (EmONC), which includes basic procedures (antibiotics, oxytocics, anticonvulsants, manual removal of placenta, assisted vaginal delivery, neonatal resuscitation) and advanced functions (blood transfusion and cesarean section).

Focusing on a hospital setting ensured frequency of interactions between care-users and providers, capturing a range of experiences—positive and negative—at the health facility interface. Through an output-based aid program ongoing at the time, the Kenyan Government identified the county as one with considerable inequities in service experience and with stigma toward poorer women (32). This site, north of Nairobi, is primarily agrarian with small shops and businesses. Care-users' homes range from shanty tin-roofed dwellings and multistory cement buildings near the town center to basic mud-and-cement structures in villages. Care-users received preventative and basic curative health services from CHWs working voluntarily in the public sector with intermittent support from non-governmental organizations. CHWs, at the time of this study, worked within community units that served catchment populations of ~5,000 (31).

### Sampling and Participant Selection

This study purposively samples a range of perspectives in a peri-urban setting (within 5-10 km radius of the sub-county hospital) to develop a contextualized model of how trust is created or undermined in maternities. Participants, selected for their ability to provide information on their perceptions of trust in maternities, were sampled from care-users (hierarchical base) over two phases before eliciting perceptions of providers and managers. The study sampled diverse perspectives with relatively open inclusion criteria. Inclusion criteria for care-users were being an adult 18 years of age or older (or living as an emancipated minor with a child), having recently had a child or being pregnant, and residing geographically near or far from the sub-county hospital. Inclusion criteria for providers and managers were working in the public sector sub-county hospital or county health teams or in varied positions in the health service delivery hierarchy. The only explicit exclusion criterion was that care-users, providers, and managers residing or practicing outside the county were ineligible.

In Phase One of the study, researchers conducted FGDs (*n* = 8) with a total of 70 care-users to understand trust determinants from a group-normative perspective. Care-user FGDs were carried out with women who had recently given birth (WRB) within the last year, whether in or out of the hospital (four groups); with pregnant women, including first pregnancies (two groups); and with male partners of women who had delivered in facilities within the year (two groups). In Phase Two, researchers conducted 33 IDIs to understand trust determinants from individual perspectives. IDI participants included women who had recently given birth (WRB) in facilities within the last 6 months (*n* = 16), different cadres of providers in facilities (*n* = 11), CHWs (*n* = 4), and managers (*n* = 2). Providers and managers interviewed included nurse-midwives, doctors (clinical and medical officers), the maternity nurse-in-charge, facility matron, and county public health officer. Providers and managers had varied experience working in their current role, ranging from 7 months to 30 years; (nurse-midwives tended to have the most experience) and were mostly women (except one clinical officer and two CHWs). Sample characteristics of our study's care-users, including age, education, marital status, parity, and occupation, are detailed in a prior publication that draws on the same sample and explores the meaning and relevance of trust within the setting (7). Care-user FGD and IDI samples were of similar age (between 18 and 37 years), faith (Christianity), and were mostly married, had at least one child, and worked outside the home. IDI care-users were more educated at the secondary or higher level compared to men and women FGD participants. Sixty-six percent of WRB in our overall sample had delivered in a facility.

Sample sizes were informed by theoretical saturation, a sampling process whereby researchers collect data to elaborate on theoretical categories until ‘no new properties' emerge (33). Prior qualitative studies that used theoretical saturation suggest that ~25-30 IDIs and 2-3 FGDs per group type, each with 8-10 participants per group, are sufficient (34, 35).

### Data Collection

Prior to data collection, the lead researcher, a South-Asian-American woman, familiarized herself over a couple of months with the geography, norms, local culture, and organizational structures involved in maternity care in Kenya. Given the study's central topic of trust, it was important to consider potential limitations associated with participants' willingness to engage in open discussion, such as language and contextual norms in community and facility settings. The study team remained engaged with the study site intermittently over about 2 months of data collection, including a week-long presence by the lead researcher in the maternity interviewing providers and managers. During this time, she drew upon her own observation of how texts, standards, and facility structure affect provider trust relationships vis-à-vis diagramming, note-taking, and asking for direct explanations within interviews.

Recruitment involved sub-county, facility, and community gatekeepers. CHWs living in the peri-urban catchment area recruited care-users: FGD participants based on group criteria and WRB in the last 6 months identified through sub-county hospital record review. Nearly all potential participants who were approached agreed to participate in the study. Care-users were interviewed in private, convenient, comfortable community settings or homes. FGD composition was intended to minimize power imbalances that might have stifled conversation or induced social unease in mixed groups. After the study's introduction to the maternity unit and county health team, the study team recruited providers, managers, and CHWs and conducted interviews in an office or field setting at the interviewee's convenience. FGDs were conducted in Kiswahili and IDIs in the respondent's preferred language (Kiswahili, English, or both), and lasted 1-2 h.

The lead researcher and four research assistants comprised the data collection team. Data collectors were female, held undergraduate diplomas in the social sciences, had qualitative data collection experience, and participated in a 4-day training covering the study's conceptual background, methodology, objectives, and protocol. Before Phase One, we pre-tested the FGD instrument at the home of a community liaison in a Nairobi neighborhood with six women who had recently given birth at a comparable hospital. Following the pre-test, we adjusted the FGD instrument language and minimized leading questions. The training emphasized practice interviewing, moderating, notetaking, making field notes, and debriefing daily. Open-ended, theoretically informed interview guides evolved over the course of inquiry. For example, learnings from Phase One informed the refinement of Phase Two's IDI guides. Care-users were asked about trust in providers and health facilities, variation and change in trust, actual vs. ideal maternity experiences, and free maternity care. Providers and managers were asked about the same topics and additionally, in accordance with the institutional ethnography approach (28), were asked about their relationships with facility staff and supervisors, and about professional practice guidelines.

### Analysis

FGDs and IDIs were digitally audio-recorded, transcribed, and translated into English; non-verbal cues were documented in interviewer field notes, which (as aligned with the recordings) were integrated into the finalized transcripts. Each transcript, accompanying field note, and recording was reviewed by at least two people to ensure quality translation of text for meaning, tone, and non-verbal cues. The research team reflected on their impressions from each interview through written field notes summarizing their perception of the interview quality, a description of the location and atmosphere, and any circumstances that may have affected the conversations. Transcript data and summary reflections contributed to the write-up of expanded field notes (27).

Our textual analysis was inductive, using grounded theory principles and the constant comparison method where the data collection and analysis follow an iterative process of informing each other and building adaptively on prior knowledge (27). We used Atlas.ti to analyze transcript data for factors determining trust in maternities. A single coder applied an inductively derived codebook in a deliberative process with the local Heshima Project team's feedback, and coded categorized text into thematically cogent areas (33). We used memo-writing to articulate codes and themes and to evaluate linkages between themes as potential trust determinants. The analysis drew on participants' responses to questions that asked explicitly about trust determinants, including “Why did you feel you could trust (or mistrust) your doctor/nurse/CHW/TBA?” and “How do you know you can trust the health system?,” followed by questions that probed on these experiences (actual or ideal) and elicited narrative examples of how they affected trust. A similar approach was used to elicit responses at the provider/manager level about trust in connection with inter-professional relationships and practice guidelines. The first author convened co-authors in an iterative process to describe and cluster themes into a final determinants framework.

The lead researcher applied memo-writing at all analysis stages to facilitate reflexivity and stimulate insights about the data (33). Memos allowed for concurrent exploration and triangulation of themes across sub-group standpoints, in accordance with institutional ethnography and appreciative inquiry (28, 29). Interpretations of the results were shared as a part of broader dissemination of Heshima Project findings to policy and program stakeholders.

### Ethics

This study received ethical approval from the Population Council Institutional Review Board (Protocol 517) and the ethics review committee of the Kenyan Medical Research Institute (KEMRI) (SCC 288). Before obtaining written informed consent, data collectors explained the study goals, described risks and potential benefits of participation, assured confidentiality in reporting de-identified responses, and emphasized the voluntary nature of the study and the right to decline participation. Compensation of 200 Kenyan Shillings (~2.50 USD) was offered to participants to cover either transportation cost or refreshment. Data were de-identified and stored on password protected computers, and hand-written notes collected were stored in locked cabinets in the PC-Nairobi office or with the lead researcher.

## Results

**Figure 2:**
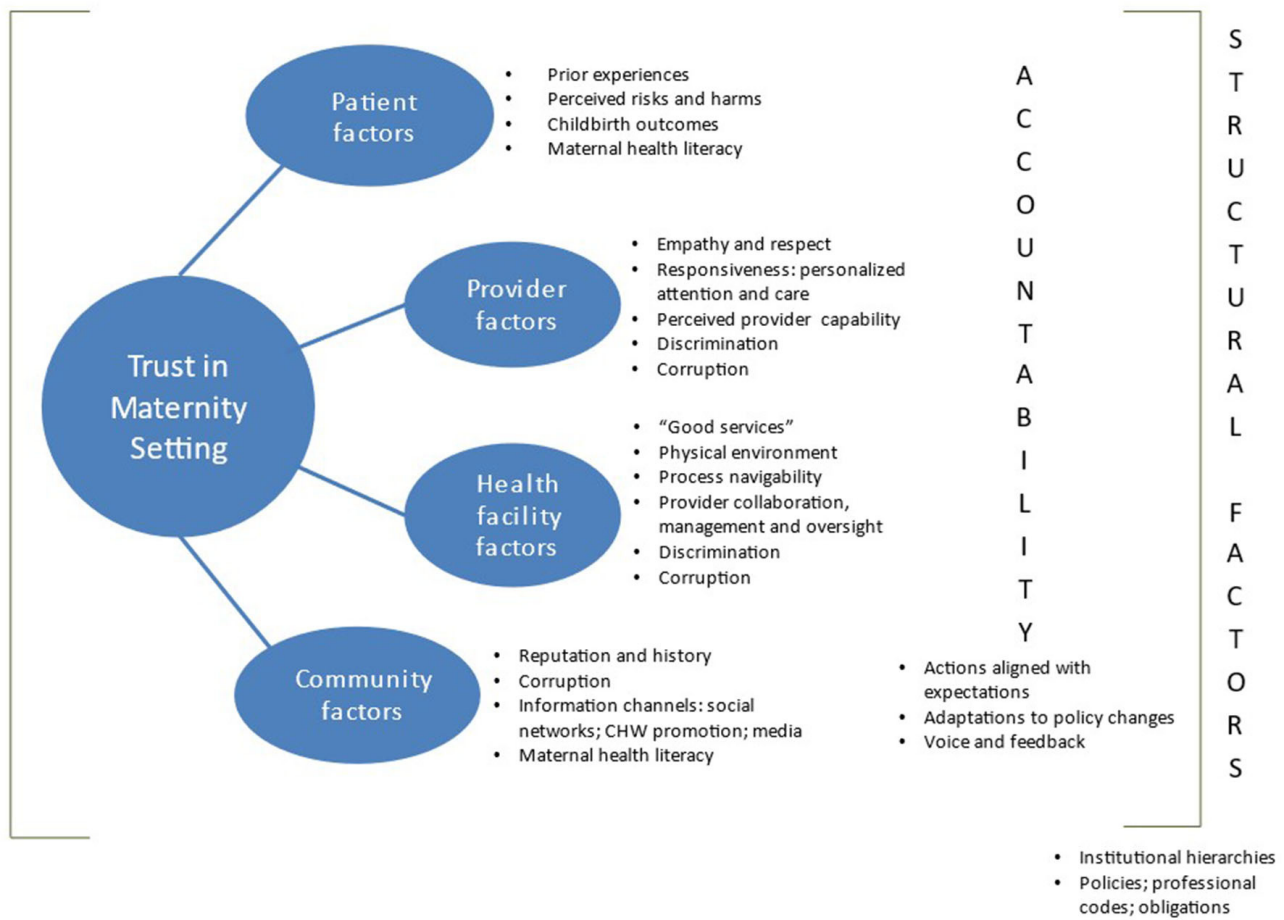
Trust determinants framework in maternity setting.

Care-user, provider, and management perspectives indicate that determinants of trust in a maternity setting can be classified broadly into six types of factors (where each type collects a cluster of factors that determine trust in the sense that they can positively or negatively influence it): patient, provider, health facility, community, accountability, and structural (Figure 2). Care-users' perspectives on interpersonal and impersonal trust are affected by the first five types of factors. Provider and management perspectives predominantly agree with care-users on these factors, add maternal health literacy as a further patient factor, and identify the sixth cluster: structural factors that underlie and affect impersonal trust in a facility.

Many of the factors that we describe here as trust determinants can also operate, through pathways other than by influencing trust, as facilitators and barriers to a good maternity experience. We here describe these factors specifically as trust determinants because our analysis identified them in participants' responses to questions that asked explicitly about trust determinants, as described in the Analysis section above.

### Patient Factors: Prior Experiences; Perceived Risks and Harms; Childbirth Outcomes; Maternal Health Literacy

#### Prior Experiences

Care-users and providers concur that the quality of a prior care experience at a facility influences trust. For many WRB and male partners, multiple births at the same facility reflect and positively determine interpersonal and impersonal trust.

“*The first childbirth went well and so did the second. The doctors present were different for each of the births…that says something about that hospital and the doctors working there.”* (Male partners, FGD).

In contrast, negative experiences and outcomes led some women to feel that their trust had been broken, deterring them from seeking care at the same facility. Negative prior experience affecting trust may pertain to either the facility or a specific provider and could occur within a single encounter or multiple facility visits.

#### Perceived Risks and Harms

While feared physical and psychosocial harms range from going “under the knife” (cesarean section) to abuse in facility-based care, women and communities describe perceived risk of infection (e.g., HIV) as affecting trust. Perceived infection risk emerged through comparisons between facility-based and home deliveries, across both individual and group-normative perspectives. Providers describe women bypassing proximate hospitals they associate with harms for similar-level alternatives farther away.

“*We even have mothers who come from Nairobi, they're neighbors to a tertiary hospital but they don't want to go there. They say we give better services, so they come.”* (Nurse-midwife, 6 years at facility).

#### Childbirth Outcomes

Care-user and provider perspectives show that positive childbirth outcomes increased trust. Specifically, these outcomes include successful management of complications and mother's prognosis, the birth of a live and healthy baby, and the ability of mother and newborn to return home in good health and without delay, which is important for women who describe needing to resume gender-specific home responsibilities. Negative childbirth outcomes, including newborn death and newborn and maternal morbidities, undermine trust. Women tend to discount their own outcomes in deference to their newborn's (“*I have faith… even if you mistreat me…the baby will be well”*). Community and provider perspectives concur that poor outcomes may have broader implications for facility reputation through co-occurrence with prior negative experience, jointly undermining trust for women and their families and communities.

#### Maternal Health Literacy

Providers consider maternal health literacy to be a factor in determining trust. “Maternal health literacy” emerged as an analytic code to reflect care-users' knowledge about the pregnancy-maternity continuum, including antenatal care, demystified understanding of facility delivery, specific labor and delivery procedures, rights and obligations of patients and providers in maternity setting, postnatal care, and family planning counseling. Providers generally attribute negative perceptions of care and reduced trust to limited maternal health literacy. Many describe how a lack of birth preparedness and not knowing what to expect during labor leads to mismatched expectations upon arrival to the facility—this often leads to negative perceptions of care, thereby diminishing interpersonal and impersonal trust. Lack of awareness about distinct provider cadre roles may undermine interpersonal and impersonal trust.

“*Some mothers come without knowing the role of a nurse or the role of a doctor; they don't know the difference. So sometimes they accuse the nurses for things beyond our capability… it's like the community is against us…”* (Nurse-midwife, 10 years at facility).

### Provider Factors: Empathy and Respect; Responsiveness; Perceived Capability

#### Empathy and Respect

Care-users and providers characterize empathy and respect together in terms of the “welcoming” nature of providers and staff, general “kindness,” “courteousness,” “friendliness,” and being treated “as a human.” Often, the social cognitive processing of these attitudes occurs via emotional cues triggered by providers' first interactions with a laboring woman. Providers and managers concur with care-users on the importance of interpersonal dynamics, and elaborate on how they try to show empathy and respect in their approach by explaining labor progression and procedures so that laboring mothers do not feel alone. WRB, pregnant women, and male partners elaborate on empathetic care using language of “good hearts” and “bad hearts.” Care-users see providers with “good hearts” actively exhibit caring and fair (non-discriminatory) behaviors that invite greater interpersonal trust in providers. Those with “bad hearts” discourage trust by behaving in ways that care-users see as impatient, insensitive, “rude,” and uncommunicative, by asking for bribes to “hasten” services, by “chas[ing] women away,” and by committing verbal or physical abuse.

Beyond overtly dismissive speech and attitudes and abusive behaviors, indirect forms of abuse, such overhearing degrading conversations by providers and trainees about women in their care, also affect trust.

“*They look at you and laugh. (*Probe: *how does that make you feel?) (*Chorus: *bad…afraid). Sometimes they speak in English, assuming you do not understand, and they make you feel very bad.”* (WRB not in facility, FGD).

While all providers and managers express intentions to provide empathetic care and subsequent trust, their mental fatigue from working in maternities results in the opposite. Providers refer to instances of transferring mental fatigue onto patients by carrying out harsh actions, which undermines care-users' interpersonal and impersonal trust.

“*It is complicated …the midwife works under pressure. You [the midwife] are alone and have to handle all those cases and out of that tension, you want the mother to behave right the way so you kind of end up shouting at the mother… She never found a calm midwife, she never found someone to handle her the right way.”* (Matron).

#### Responsiveness: Personalized Attention and Care

Women describe how provider responsiveness affects trust in terms of a nurse's or doctor's willingness and ability to limit “pain and suffering” during labor. Provider engagement in counseling signals interest in their patients, dispels fears around the delivery process, and inspires confidence. Care-users understand provider responsiveness through continual care, open dialogue, and attentiveness. Providers further consider nurse-patient relationships as influential in determining trust because nurse-midwives are the first to interact with and examine women, monitor labor, conduct normal deliveries, and provide continual care. Nurse-midwives reiterate that positive attitudes demonstrated through first impressions, verbal and facial gestures, and non-irritability signal a willingness to help. Providers understand caring as expressed in open, lively, and proactive communication.

CHWs and women report that providers' inactive listening undermines trust by discounting patients' needs, signaling disrespect, and fostering neglect. Care-users describe “feeling ignored” at points in the maternity process; these instances reflect provider delay and non-response to calls for help that led to women delivering alone or with the help of other (non-clinician) women. Women ascribe non-response to nurses “just chatting,” “sitting idle,” “too busy,” or to doctors' refusal to attend calls. In some cases, care-users' perception of systemic neglect and lack of timely response stems from witnessing others being ignored. While providers concur with care-users around inattentiveness as undermining trust, they ascribe non-response to mental fatigue due to heavy workloads and diversity of tasks in high-pressure maternities. Mental fatigue is predominantly felt by nurse-midwives and junior doctors (clinical officers and medical officers) and affects professional functioning and personal life.

“*With big workload, you are not able to handle that patient on a one-to-one basis. You are in a hurry to finish with one so that you go to the next [woman], so you don't get a good relationship.”* (Nurse-midwife, 22 years at facility).

“*You are educating, you are examining, you are delivering… all those things in the maternity! […] In the morning, I am full of strength and can do it, but when it reaches a certain hour… We also have our families. At the end of it all, you go to your family - your kid needs your support... at the end of the day, you are burned out.”* (Nurse-midwife, 8 years at facility).

#### Perceived Capability

Perceived capability of providers refers to the symbolic importance of knowledge, qualifications, and experience for the trustworthiness of formal maternity care providers. Care-users and providers felt that perceived capability contributed to both interpersonal and impersonal trust. Statements by care-users like “*s/he was skilled and knowledgeable*”, “*he is the one that knows*”, “*I can't know… just go there and listen to what they [facility-based providers] tell you*,” reflect an underlying trust in providers' expertise. Perceived capability reflects not only technical competence, but also years of experience and the way providers perform their work. In cases of unpredictable pregnancy complications, the perceived expertise of facility providers (compared to traditional birth attendants) fosters a sense of trustworthiness.

“*The difference between a TBA and a doctor is… a TBA does not know how to do a caesarian section. If you need a cesarean section, the TBA may not know what to do and maybe she could just keep you there in pain, but if you go to deliver at a facility, the doctor will examine you and know if you will need to go to the theatre.”* (RDW in facility, FGD).

### Health Facility Factors: “Good Services”; Physical Environment; Process Navigability; Provider Collaboration and Oversight; Discrimination; Corruption

#### “Good Services”

Care-users and providers repeatedly describe facilities' reliability in delivering “good services,” including during emergencies, as influencing trust. Larger hospitals (compared to smaller facilities) are perceived to possess a comprehensive set of maternity services that enable greater systems trust. The phrase “good services” refers to the overall facility capacity—enough qualified providers and adequate material resources to conduct vaginal and cesarean births. “Good services” reflects facility functioning: a steady flow of resources enabling the delivery of appreciable levels of maternity and ancillary care. Basic resources (e.g., hot water, electricity, and ambulances) and maternity-specific supplies and equipment (e.g., blood transfusion capacity, an operating theater, drugs, and cotton wool) are considered prerequisites. Typical ancillary care described by women (individually and collectively) includes the provision of warm foods and teas after birth as well as washing services for soiled clothes. Stocking medicines is integral; trust arises only when the burden of purchasing drugs does not fall on a woman or her family. Likewise, without independent facility financing for supplies, procurement delays occur, diminishing care-user perceptions of “good services.”

#### Physical Environment

Women and communities describe congestion, cleanliness, and hygienic conditions as influencing their trust in the facility. Providers and managers note that beyond cleanliness and lack of a maternity-specific operating theater for cesarean sections, bed shortages and congestion affect trust by limiting providers' ability to maintain confidentiality. Space shortage is cited in connection with doctors' rounds, where routine efforts to maintain patient confidentiality (such as not disclosing HIV status) are undermined by discussions among doctors, students, and nurses about treatment. Providers and managers also describe deficiencies in supply chains for material resources (e.g., drugs, gloves, boots for the delivery room, obstetric equipment, cotton wool) as undermining trust by hindering the provision of quality maternity care.

#### Process Navigability

Many care-users felt the need for streamlining and simplifying the processes facing women in maternities. Paperwork, communication across departments and providers, payment and financing, queuing, and frustration at being shuttled around affect trust. Logistical challenges occur when diagnostic tests are administered elsewhere. Male partners or accompanying family members may be transferred between payment, procedures, and additional material resource purchases, leading to delays in care. Like perceptions of “good services,” process navigability influences care-users' impersonal trust in facility functioning.

“*R4: There is also the system of how payments are made...When you go to the maternity if you are taking your wife to deliver, you still have to buy these ward forms. (R3): You have to go back and queue to pay for it… If your wife was in labor pain, she is given drugs which you have to pay for. After you pay you go to another queue to collect those drugs. That system is too long. It would be better if they could shorten those systems.”* (Male partners, FGD).

#### Provider Collaboration, and Oversight

Because maternity care demands a range of skills and cadres of health workers, care-users and providers see collaboration amongst providers as a determinant of impersonal trust. Providers see collaboration as embedded in working relationships, and care-users' perspectives invoke the congruency of efforts witnessed over the course of a woman's stay in the wards.

“*We have to work as a team. You can't manage a patient alone… you communicate with the others, work together, and have a good relationship…When you need help… you call for help and everyone stops whatever they are doing and comes to your rescue. You do everything for the mother.”* (Nurse-midwife, 3 years at facility).

“*They [providers] made me happy. I saw them love each other, talk to each other well, and if a patient encountered a certain problem… they call the doctor and tell her that a particular patient is like this…she comes -because the other cannot handle- and helps out.”* (WRB, 25 years, IDI).

Contrastingly, WRB report that witnessing discord or negative tone among providers may diminish trust in the facility. Situations where coordination supports the mother but not the newborn (e.g., newborn death) were also described as undermining trust.

Care-users describe how providers appeared overworked, and how unsupported provider behavior can lead to diminished patient trust. They also express the need for supervisory oversight to prevent rude/harsh provider behaviors.

“*(R7): I don't know if it is that the nurses are overworked and tired, since they are rude every time you get there. (R1): I think they just don't love their jobs. It's as if they are tired of it and just go to work each day as routine.”* (WRB in facility, FGD).

Providers and managers agree that trust is undermined by limited staffing and overworked staff experiencing mental fatigue, particularly those on night shifts covering more than one ward. Many providers describe the need for more timely and responsive supervision to mitigate their fatigue and foster better collaboration.

#### Discrimination

Discrimination, understood by all participants as unjust differential treatment, undermines trust and discourages future maternity care-seeking. Women and community members describe experiencing discrimination based on wealth, age, and parity. For example, voucher users, perceived as poor, commonly experience longer wait times compared to those who pay cash. Although first-time mothers generally experience congenial treatment by providers compared to older, higher-parity women, IDIs reveal provider prejudices regarding women's fertility and birth spacing.

“*If [providers] see a young pregnant girl… they [ask] whether it is education they want or to get pregnant…. [or]... Older women giving birth also fear delivering in the hospital... They go to deliver their 7*^*th*^ *child… doctors ask, ‘are you delivering - isn't it your child's turn?”* (CHW, male, IDI).

Although providers and managers agree that discrimination affects care-users' trust, they deny engaging in discrimination themselves.

#### Corruption

Many providers concur with care-users that the existence of “shortcuts” or the practice of “giving something small” as part of facility culture undermines trust, though they do not admit to personally accepting informal payments.

“*I attend to patients without shortcuts… it is good to be genuine. If there is something to be paid, let them use the right channel, let them pay thorough the cash office and given the receipt, so nobody pockets their money… patients are innocent. Sometimes they come and whoever is attending them asks for some money; nobody will know because they are alone in the room... Patients should know their rights and that if they pay, they should be given a receipt.”* (Nurse-midwife, 10 years at facility).

This quotation endorses an ethical demand that providers refrain from “pocketing” bribes, but also places responsibility on care-users to know where the legitimate payment channels are, and on health facility management to prevent corruption from occurring by providing payment receipts.

Health managers reflect on the duality of corruption as a health-facility trust determinant. They see bribes as socio-culturally commonplace, suggesting that incentives weigh against reporting bribes: bribe acceptors benefit financially, and payers experience more efficient care.

“*In a case of corruption, many (patients/community) end up pleading for the staff helping them – ‘you want to punish them and they were helping me?! Next time I come, hata nisaidia (s/he won't help me).'… If I give this little money here I won't pay the big money there. There is suspicion. I would rather give you two hundred shillings here rather than pay one hundred shillings at the registry cause I think it won't be one hundred shillings there.”* (Matron).

Despite the commonality of informal payments in other facets of social and political life, many participants found this practice disruptive to impersonal trust in maternities, where women's vulnerability is high.

### Community Factors: Facility Reputation and History; Information Channels

#### Facility Reputation and History

Care-users and providers indicate that a facility's reputation and history influence impersonal trust. Women and communities elaborate on how negative or positive facility reputations are created through an accumulation of individual experiences shared within communities over time. First time pregnant women noted reputation as particularly influential on their sense of trust.

“*Maybe you have heard that this woman went there, had a complication and the doctors didn't know it. If you hear of two or three such cases, you will fear.”* (1^st^ time pregnant woman, FGD).

The frequency and nature of communicating experiences may affect how facility reputation is built or broken as well as reflect general trusting or mistrusting attitudes in the community. Trust is also affected by the social history of the county, where men and women value and act on accounts provided by their families, neighbors, and other community members. The collective memory of detention for inability to pay prior to the free maternity policy underlies participant perceptions of discrimination and residually affects trust in this sub-county.

#### Information Channels: Social Networks; CHW Promotion; Media

Care-users and providers describe social networks as the most common information channel influencing care-users' interpersonal and impersonal trust. Informal conversations among women, male partners, close family, friends, and neighbors affect trust and mediate care-seeking decisions and behaviors, birth preparedness, and treatment norms.

“*I trusted it because my neighbors had been treated there. They had told me how well they had been attended to there, the services offered there and so I decided to take her there too.”* (Male partners, FGD).

“*(R1): You get prepared psychologically [from conversations]…If you get a certain feeling while at home, you may not be sure what it is. But not if one who gone through it [childbirth] already told you of it. (R2): It helps you to be prepared.”* (1^st^ time pregnant women, FGD).

Conversations occur in antenatal clinic waiting areas, “*chamas*” (social and financial support groups), community events, and social gatherings. While network-based learning is helpful to women, providers felt that social networks may undermine trust so far as they perpetuate misunderstandings of facility care.

Care-users and CHWs primarily describe CHW promotion as a second information channel affecting trust, so far as CHWs are accessible information sources within the community. Although many WRB and male partners living in the peri-urban county value CHWs, numerous care-users said they could not locate and use them effectively. FGDs suggested that not all community members had prior interactions with CHWs in the area before this study's recruitment. Exposure to and familiarity with CHWs and their role may affect the believability of CHWs' messages. Care-users and CHWs felt that a CHW's links to the facility and other health areas, as well as his or her involvement in community life (church, schools health campaigns, chief's *barazas* [meetings], social rites of passage), render him or her a reliable information source.

“*I meet pregnant women…and they tell me how they are faring on… I tell them to get safe delivery at the hospital so that they can avoid the risks which can occur when they are delivering back at home…..We are free with them… my relationship is good because I am able to reach them and we talk... They have my number, they can call.”* (CHW, female, IDI).

Care-users' trust in maternity settings draws to a smaller and variable extent on media-based publicity. Media—primarily radio—emerged as an avenue for praising or shaming facilities. Some respondents suggest that shaming motivates facility management and providers to change service processes and behaviors; others feel that positive media portrayal of public facilities promotes a trustworthy image.

### Accountability Factors: Actions Aligned With Expectations; Adaptation to Policy Changes; Voice and Feedback

#### Actions Aligned With Expectations

Care-users and providers describe responsible parties' delivery of promised actions as enhancing impersonal trust in facilities, which is linked to trust in broader social institutions. While some care-users describe the newly elected government's efforts to address corruption cases, all mention the importance of transparency in responsibly deploying public funds toward their intended use. In health facilities, this alignment of action speaks to two expectations: receiving care worth what one pays for, and providing free care to those who cannot afford to pay. Examples of expectation-action alignment include reductions in discrimination and corruption or improvements in “good services.” In maternities, expectations and actions revolve around the quality of interpersonal interactions and responsiveness to care-user needs.

“*The patient is never at fault… they should not shout at me… The moment you go there [facility], you are innocent and they [providers] should treat you. It's the doctors with a problem… they need to change.”* (1^st^ time pregnant women, FGD).

This sentiment captures the inherent vulnerability of laboring mothers, recognizes the power wielded by nurses and doctors, and holds that women deserve to receive treatment without blame. Although this proposition drew consensus across WRB, pregnant women, male partners, and CHWs as what “ought to be” standard practice in maternities, the underlying tone suggests a discrepancy between ideal and actual experience. Care-users convey that when individual patient-provider interactions meet their expectations, interpersonal trust is elevated.

For providers and managers, alignment of action with expectations, as related to trust, covers managers' responses to nurse and doctor requests, and government provision of adequate supplies and allocation of sufficient human resources. At the health systems level, the expectation-action relationship as related to trust is rooted in user perceptions of the MOH's role in effectively implementing reproductive health policies.

#### Adaptation to Policy Changes

Moments of policy change prompt providers and managers to reflect on and evaluate how accountable their facilities are, thereby affecting care-user trust in the maternity setting. First, national devolution to county governments was understood by providers/managers and care-users to affect roles and responsibilities within the Kenyan health system, which has repercussions for accountability and trust. Second, participants' reported initial responses to Kenya's free maternity policy illustrate how policy rhetoric aimed at benefitting the poor must be incorporated into a facility context that is already subject to a normative understanding of service delivery and experience. Skepticism across care-user and providers about the implementation of free maternity stems from mismatched expectations and action in past social programs (e.g., free education) that fell short of rhetoric (e.g., parents required to pay administrators to enroll children). As an example of the undermining of care-users' impersonal trust, community members expressed a fear of further stigmatizing the poor, given the plausible expectation that the facility would be unable to adapt to the free-maternity policy.

“*I think that [free maternity care] will help in a big way for woman who are underprivileged…But also, I feel like the services could deteriorate…Because at the maternity, they will feel like they are doing charity work for you…I feel it will be worse… someone could insult you now during delivery when you pay for the service. So when they know you didn't pay, won't they insult you more? I personally feel it will be worse; I am even scared of going there now.”* (WRB, 27 years, IDI).

#### Voice and Feedback

Care-users and providers indicate that dialogue and the inclusion of their collective voices, as a part of accountability processes in maternities, affect impersonal trust. On the one hand, cultural deference to medical expertise (“when the nurse tells you anything, you should do it”), and fear of repercussions on future access or care (“you do not want to be sent away”) that compel care-users and their families to stay silent in facilities, undermine trust. On the other hand, all participants value community voice as integral to maintaining accountability and enhancing trust. Participants collectively explain that women should be able to ask questions, demand their rights, and report complaints before, during, and after their childbirth experience.

“*Patients should not fear doctors. They should desire to know, ‘how is my health?… Patients have a right to be educated…that way you build confidence and trust with the provider.”* (CHW, female, IDI).

This CHW's reflection captures the essence of a publicly accountable health system where providers respect and respond to patients' queries. Dialogue as a precursor for care-user trust in maternity settings reflects the entitlements of taxpayers as beneficiaries of government sector services (“*these hospital workers are paid with our taxes, right?*” —Male partner). The inclusion of community voice emerges during discussions of citizens' oversight roles in ensuring acceptable governance of social institutions (including hospitals) given taxpayers' contributions, principles of democracy, and a people-centered constitution.

Provider and management perspectives corroborate care-users' association of accountability with trust by reflecting on intra-facility feedback where facility management collects feedback from patients through routine mechanisms (e.g., customer care desk, a suggestion box, recently established bi-annual quality assurance audits). Yet, delays in sharing findings with providers reduce accountability to health workers, affecting care-users' impersonal trust in maternities.

“*There is somebody who allocated to quality assurance at the hospital…if you don't get feedback, you don't know where you are going wrong, where you need to improve. I've only been given feedback once…”* (Nurse-midwife/in-charge, 11 years at facility).

Providers further note, as related to trust, the importance of engaging voices of frontline providers in feedback cycles to improve facility accountability. Some providers, particularly nurse-midwives, express the view that their perspectives ought to be valued within the quality improvement process.

*Our in-charges should listen to us. We cannot always be right, but we have a point…if we are expected to serve patients well… somebody somewhere should listen to me*. (Nurse-midwife, 8 years at facility).

### Structural Factors: Institutional Hierarchies; Policies and Professional Codes

Provider and management perspectives on their relationships with each other and patients, as well as on facility policies, guidelines, and norms, suggested that embedded power dynamics in maternities are structural factors that indirectly affect care-users' impersonal trust. Providers describe power imbalances within provider-provider and provider-care-user interactions as reinforced by normative codes that affect care-user experience and subsequent trust.

#### Institutional Hierarchies

Institutional hierarchies, including facility and provider-patient hierarches, affect inter-provider power dynamics, which indirectly determine care-user trust in maternities. Health providers, primarily nurses or interns, experience professional hierarchies as norms of deference to authority and as feelings of disempowerment when berated or dismissed by senior consultants.

“*The doctor comes to see the patient – ten minutes or so – and then he or she is gone… the nurse can't make all decisions, but at least they [doctors] should listen to us.”* (Nurse-midwife, 8 years at facility).

“*Doctors have that ego, ‘we know how to diagnose, we know how to do things right.'... There are some [senior consultants] who tell the nurses off in front of patients and all of us - which is not right [respectful]. But there are some doctors who actually [show respect]...It is not age -related - it's just that doctor-nurse relationship.”* (Medical officer intern, 1 year at facility).

These facility hierarchies are transferred onto patients *via* provider-patient hierarchical norms that affect care-user trust. Providers experience these institutional hierarchical norms as gendered structures, where managers internalize their supervisory roles and nurse-midwives internalize their role toward patients as “superior.” This is evident among all providers in their use of the term “cooperation” referring to how well patients obeyed providers' instructions during labor and delivery. When patients “cooperate,” they have positive experiences—according to providers—which enhances impersonal trust. When this cooperation disappears or is “complicated” (i.e., patient does not follow instruction or communicate), some providers blame patients for negative outcomes, which undermines care-user trust.

“*But if she is uncooperative, she will just push and push until the whole place is just been swollen and may be the head has been mangled or the baby will just die there because she is not cooperative…”* (Nurse-midwife, 30 years).

“*The way you [patient] come and present yourself, guides me on how I treat you. If you keep things to yourself I will leave out some things [e.g. history]….so if I make I mistake, I made it cause you [patient] led me to that.”* (Clinical officer, 7 months in facility).

### Policies and Professional Codes

Hierarchical interactional norms often follow official documents: policies and professional codes that contextualize practice and indirectly affect care-users' impersonal trust through health facility and accountability factors. Providers elaborate on their fiduciary roles, decision-making, and behaviors in accordance with Kenyan policies, professional codes of ethics, national guidelines, and service charters. Managers additionally describe guidelines for money management, dispute resolution, and disciplinary action. Though some of these documents—the Nursing and Citizen's Service Charters—are posted in waiting areas, provider reflections suggest that their influence on patient-provider interactions is mixed.

“*It's useful because it reminds us of what is expected of us and what is expected of the patients also… Most [patients] don't even read it …they are pre-occupied - they don't see it... It can be useful because they can see there is something expected of them*.” (Nurse-midwife, 10 years at facility).

“*If we had time [for counseling] we would use it [nursing and citizen's charter], but here rarely do we use it.”* (Nurse-midwife, 3 years at facility).

While providers agreed that service charters ought to be used as a trust-enhancing mechanism, hierarchical sub-texts affect the way in which they are used, and these hierarchical sub-texts can be masked in seemingly neutral language of patient “cooperation.” Providers recognize patient rights, but express frustration about a lack of documented provider protections. In instances where there is a poor maternal outcome (e.g., a maternal death), individual nurses may suffer the blame of communities, which manifests as diminished impersonal trust.

## Discussion

The qualitatively derived multi-faceted determinants framework reveals that trust in a facility-based maternity setting is reportedly influenced by patient, provider, health facility, community, accountability, and structural factors. While there is consensus between care-users and providers around these clusters, each standpoint emphasizes different sub-themes with varied narratives of how each factor relates to others and influences trust (6, 14). For instance, a care-user's trust may be influenced simultaneously and in different ways by her perceived risk, provider empathy, the navigability of processes in maternity wards, her social network, and whether her voice is heard and responded to through reciprocal interactions with facility management and providers. While providers overarchingly agree with most trust determinants, they ascribe care-user trust to varied maternal health literacy in addition to prior individual and shared experiences at a facility. At the same time, providers and managers additionally describe the need for supervisory and facility support of their own needs, unveiling the indirect influence of institutional hierarchies and professional codes on practice norms, which translate through power relationships in maternities into care-users' experiences, thereby affecting trust. In our peri-urban Kenyan setting, care-users and providers both suggest that provider collaboration, experience of the free maternity and devolution policies, and shifting tools for respectful care (e.g., nursing and citizen service charters) can indirectly affect care-user trust in maternities.

### Trust Determinants and Respectful Person-Centered Maternity Care

Consistent with trust literature, our study suggests that patient and community determinants of trust in maternity care revolve around socio-cultural and experience-based factors including risk perception of the hospital environment, traditional norms around birth, a facility's historical reputation, and social networks. Prior experience and perceived harms associated with facility-based childbirth are consistent with determinants of trust in maternities in Thailand (26). Perceived risk in peri-urban Kenya covers both social and biomedical aspects of prior experience at facilities. Positive and negative facility-based maternity experiences, as described in extant respectful maternity care (RMC) and person-centered maternity care (PCMC) literatures (36, 37), have implications for care-seeking; our study elaborates on how these relationships can be mediated by individual and collective care-user trust in maternity care. While positive patient-facility interactions increase service use due to elevated trust generally, we saw from women's and men's recollection of past inequitable treatment (detention for inability to pay) that lower trust among historically marginalized groups deters care seeking, similar to other studies (38). Providers in our study, like those studied elsewhere, perceived bypassing of facilities in under-resourced settings as exemplifying variable trust among women seeking healthcare (12). Facility reputation, reflecting collective experience of women's social networks, was expressed across standpoints as perpetuating higher or lower trust in maternities. Some providers foresee higher salience of socio-cultural and experience-based trust determinants in the free-maternity context, where affordability is less influential.

Our study's identified health facility and provider determinants of trust align with the global consensus around the importance of technical and experiential aspects of quality care (6, 39). They also demonstrate concurrence of care-user and provider perspectives and are consistent with features of RMC and PCMC (40, 41). Provider perspectives reveal that negative patient-provider interactions often stem from mental fatigue and low staff morale caused by over-working, lack of oversight and poor environment (42). Research on similar trends observed in the RMC literature proposes solutions such as provider support policies, trainings, and infrastructural improvements among others to improve care-user-provider collaboration (43). Our findings also show that reciprocal relationships and mutual respect gained from workplace collaboration between different cadres are integral to determining trust in maternities (6). Effective collaboration in maternity care involves organizational, procedural, relational, and contextual confluence across worker actions (44). Our study, similar to others in Ghana and Tanzania, shows that team-based efforts to enable a mother to safely give birth are often hindered by health system power asymmetries that negatively affect collaboration in practice (45, 46). We found non-response of supervisors, verbalizing position-based “superiority,” and normalized verbal abuse between colleagues and toward lower-tier workers undermine collaborative spirit and women's trust. Gender offers a salient lens to interpreting institutional hierarchical norms affecting the providers' trust of each other.

### Power Structures and Trust

Our study's integration of female care-user and provider standpoints elucidates the influence on trust in maternity care of gender as, first, a social norm that dictates women's domestic roles and, second, a structural process that arises out of power asymmetries (within the patient-provider-manager hierarchy) at health facilities. From the care-user perspective, prevailing norms amongst women tend to value newborn outcomes over the quality of their own experience when it comes to trust in the maternity setting. This is congruent with gender norms in Kenya where 20% of women experience disrespect and abuse in facility-based maternity care (47) and, although male partners dominate reproductive decision-making, childbirth experience and its repercussions traditionally fall under women's purview (10). Gendered patient-provider interactions determining trusting relationships are best elucidated through the lens of female nurse-midwives in maternities given their intersectional role within professional and social-power-laden structural contexts (48). Being a provider positions a nurse-midwife at a higher power level compared to a patient but institutionally at a lower power level compared to doctors and facility managers (45). Gendered household responsibilities of female providers reflect socio-cultural pressures that affect nurse-midwives' capacity and morale (49). Structural factors that privilege doctors over nurses, exacerbated by facility trust determinants like inadequate resources, may impose mental fatigue onto birthing women by way of normalized mistreatment by providers (50, 51), with subsequent implications for trust.

We posit that policy and political contexts influence trust through accountability factors that care-givers and providers characterize within expectation-action relationships, health facility adaption to policy change, and inclusive feedback, all of which relate to the governance, provision, and use of maternity care (52, 53). In Kenya, free maternity and devolution policies induce stress on constrained environments and a limited health workforce (54). Our study findings imply, for example, that care-user trust may be undermined because the supply of nurses does not match the increased demand for maternity care. Devolution shifts expectations onto health professionals to be active stewards of facility and county financing and decision-making; lack of training in these roles may exacerbate inequities in care experienced within counties (55, 56). At the time of our study, health providers, managers and county policymakers expressed difficulty in developing a devolved county strategy to cover health facilities, community units, and their linkage. All standpoints felt uncertain of free maternity's implications for trust given the expectation for care provision on the one hand, and, on the other, anticipated increased susceptibility to mistreatment for those who had already experienced discrimination and corruption in the system. Skepticism stemmed from informal cash-exchanges experienced in health facilities that benefit the financially able compared to the disadvantaged poor and undermine trust (57). Our findings suggest that while discrimination at the health service interface may be under-recognized as problematic (58), a care-user's fear of “speaking up” to avoid harsh treatment by a provider may embody an internalization of Kenya's prior institutional culture that silenced individuals opposing authority (10).

### Strengths and Limitations

Our study's findings should be interpreted alongside its strengths and limitations. Our critical approach, explicitly triangulating distinct standpoints of care-users (individually and collectively) and providers and managers, demonstrates a robust way to understand how socio-political relationships, power, and gender affect trust. Many care-users and providers describe their study participation as a uniquely welcome opportunity to share their thoughts about an important and timely topic. One limitation is that the study is cross-sectional. While data were collected over a 3-month period for pragmatic reasons, the 2 preceding months spent establishing critical contacts within the peri-urban area, as well as detailed field notes and daily debriefs, assured that the research team ascertained meaningful data. Social desirability bias was minimized through engagement in a reflexive process and triangulation of data. To limit recall bias inherent in self-report data, we focused on recent maternity experiences within 6 months and 1 year for individual and collective perspectives, respectively. Given that this study focused on one geographic, linguistic, and sociocultural context among the many settings in Kenya, and our sample possibly excluded vulnerable women with less than primary education, its transferability may be limited. We recommend that future studies diversify their exploration of trust across regions and sub-populations. Finally, despite the time-lag between data collection and publication, the interpretability of trust determinants remains relevant and related to respectful maternity care and women-centered care, particularly as it increasingly is seen as an outcome of health systems and service quality.

## Conclusion

Drawing on care-user and provider perspectives in peri-urban Kenya, this study identifies and proposes a multi-faceted set of trust determinants in a maternity setting. The overlapping, reinforcing, and potentially opposing determinants render trust a complex but critical construct for understanding the social and political contexts of a health system. The framework's complementarity with global understandings of quality, respect, person-centeredness, and gender in maternity care, suggests that trust determinants may be transferable and warrant investigation. Further well-documented implementation and action research is needed to assess trust-building interventions in complex environments, including interventions that promote more equitable power dynamics within maternity care at multiple levels of the health service delivery interface.

## Data Availability Statement

The raw data supporting the conclusions of this article will be made available by the authors, without undue reservation.

## Ethics Statement

The studies involving human participants were reviewed and approved by Population Council International Review Board. The patients/participants provided their written informed consent to participate in this study.

## Author Contributions

PS conceptualized, designed, and implemented this study and wrote the manuscript. MM guided the conceptual process and provided substantive feedback throughout analysis, writing, and editing of the manuscript. DK supported the theoretical approach and provided feedback on analyses and iterations of the manuscript. TA and CN oversaw and supporting the fieldwork, contributed to data management, provided feedback through the analytic process, and provided local insight to the study. CW provided contextual grounding and expertise in maternal health research that strengthened the relevance of these findings in Kenya. All authors reviewed and approved the final version of this manuscript.

## Funding

We are grateful for funding from USAID TRAction (No. GHS-A-00-09-00015-00) that enabled data collection under the Heshima Project, under which data for this manuscript is drawn.

## Conflict of Interest

The authors declare that the research was conducted in the absence of any commercial or financial relationships that could be construed as a potential conflict of interest.

## Publisher's Note

All claims expressed in this article are solely those of the authors and do not necessarily represent those of their affiliated organizations, or those of the publisher, the editors and the reviewers. Any product that may be evaluated in this article, or claim that may be made by its manufacturer, is not guaranteed or endorsed by the publisher.
